# Supplementary figures and images for: Olfactory recovery following infection with COVID-19: A systematic review
Source: PLoS One. 2021 Nov 9;16(11):e0259321. doi: 10.1371/journal.pone.0259321 (PMC8577770; doi:10.1371/journal.pone.0259321)

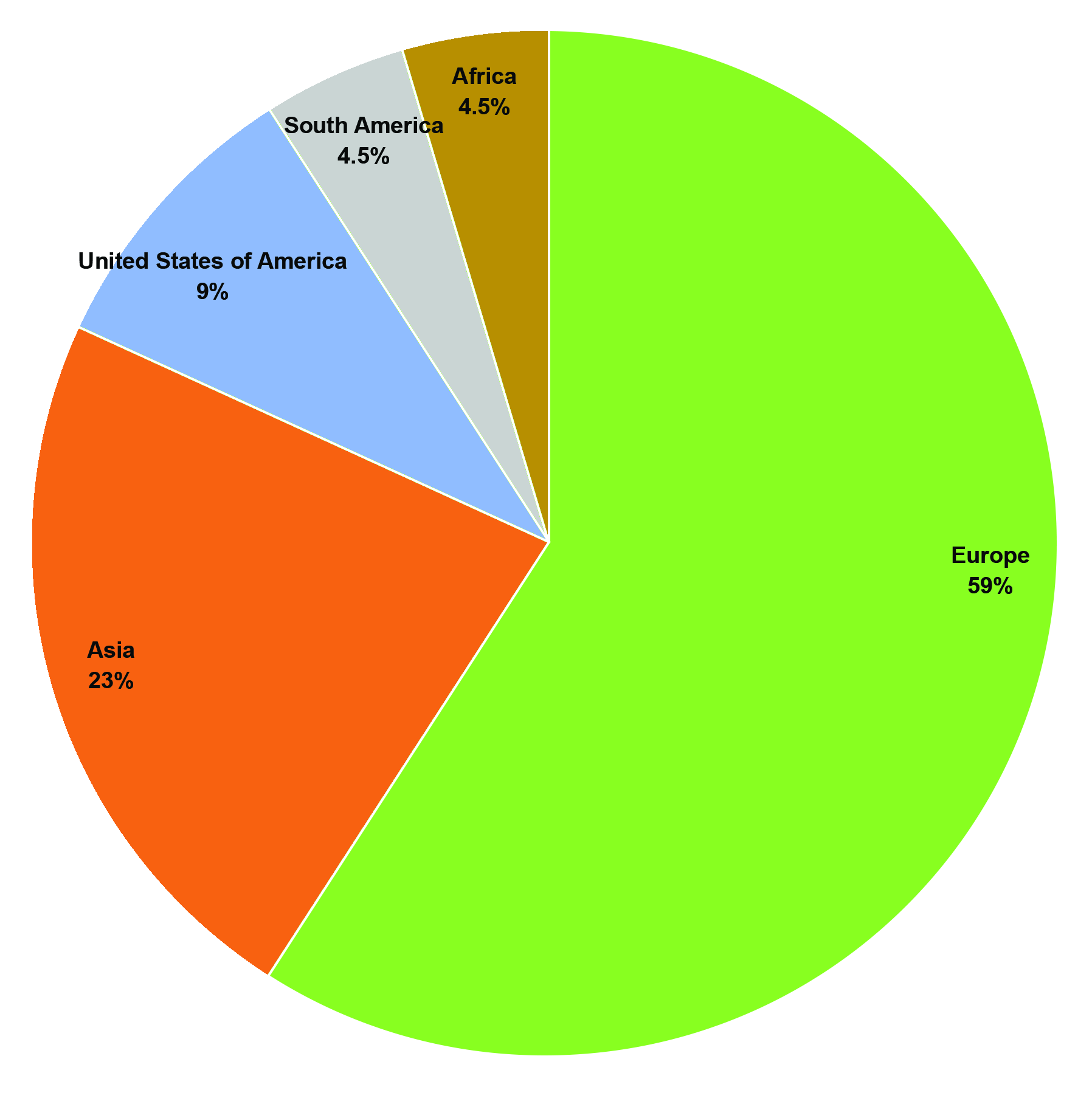

Supplement: S1 Fig — (TIF) [file pone.0259321.s001.tif]
