# Supplementary material for: Olfactory recovery following infection with COVID-19: A systematic review
Source: PLoS One. 2021 Nov 9;16(11):e0259321. doi: 10.1371/journal.pone.0259321 (PMC8577770; doi:10.1371/journal.pone.0259321)
Supplement: S1 Table — (DOCX) [file pone.0259321.s002.docx]

S1 Table. Checklist for Analytical Cross-Sectional Studies

| Study ID | Were criteria for inclusion in sample clearly defined? | Were the study subjects and the setting described in detail? | Was the exposure measured in a valid and reliable way? | Were objective, standard criteria used for measurement of the condition? | Were confounding factors identified? | Were strategies to deal with confounding factors stated? | Were the outcomes measured in a valid and reliable way? | Was appropriate statistical analysis used? |
| --- | --- | --- | --- | --- | --- | --- | --- | --- |
| Yan 2020 | yes | no | yes | no | no | no | no | yes |
| Gorzkowski 2020 | yes | yes | yes | no | no | no | no | yes |
| Dell'Era 2020 | yes | yes | yes | no | no | no | no | yes |
| Chiesa-Estomba 2020 | yes | yes | yes | no | no | yes | yes | unclear |
| Boscolo-Rizzo 2020 | yes | yes | yes |  | yes | no | yes | yes |
| Amer 2020 | yes | yes | yes | yes | yes | yes | yes | unclear |
| Lechien 2020 | unclear | yes | yes | yes | no | no | yes | yes |
| Vaira 2020-1 | yes | yes | yes | yes | no | yes | yes | yes |
| Jalessi 2020 | yes | yes | yes | no | yes | no | yes | yes |
| BrandaoNeto 2020 | yes | yes | yes | no | yes | yes | unclear | unclear |
| Renaud 2020 | yes | yes | no | no | yes | no | no | unclear |
| Asad 2021 | unclear | no | yes | yes | no | no | yes | yes |
| Biadsee 2021 | yes | yes | yes | yes | no | no | yes | yes |
| Bulgurcu 2020 | yes | yes | yes | yes | no | no | no | yes |
| Sahoo 2021 | yes | yes | yes | yes | no | no | yes | yes |
| Kavaz 2021 | yes | yes | yes | yes | yes | no | no | yes |

Table 2. Checklist for Cohort Studies

| Study ID | Were the two groups similar and recruited from the same population? | Were the exposures measured similarly to assign people to both exposed and unexposed groups? | Was the exposure measured in a valid and reliable way? | Were confounding factors identified? | Were strategies to deal with confounding factors stated? | Were the groups/participants free of the outcome at the start of the study? | Were the outcomes measured in a valid and reliable way? | Was the follow up time reported and sufficient to be long enough for outcomes to occur? | Was the follow up complete, and if not, were the reasons to loss to follow up described and explored? | Were strategies to address incomplete follow up utilized? | Was appropriate statistical analysis used? |
| --- | --- | --- | --- | --- | --- | --- | --- | --- | --- | --- | --- |
| Vaira 2020 | n/a | n/a | yes | No | No | n/a | yes | yes | unclear | unclear | yes |
| Speth 2020 | n/a | n/a | unclear | no | no | n/a | unclear | unclear | unclear | unclear | unclear |
| Konstantinidis 2020 | n/a | n/a | yes | no | unclear | n/a | no | yes | yes | n/a | yes |
| Vaira 2020-3 | n/a | n/a | yes | yes | yes | n/a | yes | yes | yes | unclear | yes |
| Iannuzzi 2020 | n/a | n/a | yes | yes | yes | yes | yes | yes | yes | n/a | yes |
| Bertlich 2021 | yes | yes | yes | no | no | yes | yes | yes | no | no | unclear |
| González 2021 | yes | yes | yes | yes | yes | no | yes | yes | yes | no | yes |
| Lechien 2021 | n/a | n/a | yes | No | no | unclear | yes | yes | no | no | yes |
| Locatello 2021 | n/a | n/a | yes | yes | yes | yes | yes | yes | no | no | yes |
| Niklassen 2021 | n/a | n/a | yes | yes | no | yes | yes | yes | no | no | no |
| Otte 2021 | n/a | n/a | yes | yes | no | no | yes | yes | yes | n/a | unclear |
| Petrocelli 2021 | n/a | n/a | yes | yes | unclear | yes | yes | yes | yes | n/a | yes |
| Raad 2021 | n/a | n/a | yes | yes | yes | no | no | yes | no | no | yes |
| Salcan 2021 | n/a | n/a | yes | No | no | no | yes | unclear | yes | no | yes |
| Ugurlu 2021 | n/a | n/a | yes | yes | yes | yes | yes | yes | yes | n/a | yes |
| Yadav 2021 | n/a | n/a | yes | yes | yes | unclear | yes | yes | yes | n/a | yes |

Table 3. Checklist for Case Control Studies

| Study ID | Were groups comparable other than the presence of disease in cases or absence of disease in controls? | Were cases and controls matched appropriately? | Were the same criteria used for identification of cases and controls? | Was exposure measured in a standard, valid and reliable way? | Was exposure measured in the same way for cases and controls? | Were confounding factors identified? | Were strategies to deal with confounding factors stated? | Were outcomes assessed in a standard, valid and reliable way for cases and controls? | Was the exposure period of interest long enough to be meaningful? | Was appropriate statistical analysis used? |
| --- | --- | --- | --- | --- | --- | --- | --- | --- | --- | --- |
| Vaira 2020-2 | yes | n/a | yes | yes | yes | No | yes | yes | Yes | yes |
| Moein 2020 | unclear | yes | No | yes | yes | yes | yes | No | yes | yes |
| Martin-Sanz 2020 | unclear | No | yes | yes | yes | unclear | unclear | yes | yes | yes |
| Eliezer 2020 | unclear | yes | No | yes | No | No | no | yes | yes | yes |
| Riestra-Ayora 2021 | yes | yes | yes | yes | yes | yes | yes | yes | Yes | yes |

Table 4. Checklist for Case Series

| Study ID | Were there clear criteria for inclusion in the case series? | Was the condition measured in a standard, reliable way for all participants included in the case series? | Were valid methods used for identification of the condition for all participants included in the case series? | Did the case series have consecutive inclusion of participants? | Did the case series have complete inclusion of participants? | Was there clear reporting of demographics of the participants in the study? | Was there clear reporting of clinical information of the participants? | Were the outcomes or follow up results of cases clearly reported? | Was there clear reporting of the presenting site(s)/clinic(s) demographic information? | Was statistical analysis appropriate? |
| --- | --- | --- | --- | --- | --- | --- | --- | --- | --- | --- |
| Klimek 2020 | Yes | yes | yes | Yes | yes | unclear | yes | yes | yes | yes |
| Janowitz 2020 | n/a | no | yes | yes | yes | yes | yes | yes | no | yes |
| Freni 2020 | yes | yes | yes | unclear | yes | yes | yes | yes | yes | yes |
| Chary 2020 | yes | yes | yes | yes | yes | yes | yes | yes | no | No |
| Sakalli 2020 | yes | yes | yes | No | No | yes | yes | yes | no | yes |

Table 5. Checklist for Randomized Controlled Trials

| Study ID | Was the randomization used for assignment of participants to treatment groups? | Was allocation to treatment groups concealed? | Were treatment groups similar at baseline? | Were participants blind to treatment assignments? | Were those delivering treatment blind to treatment assignments? | Were outcomes assessors blind to treatment assignment? | Were treatment groups treated identically other than the intervention of interest? | Was follow up complete and if not, were differences between groups in terms of their follow up adequately described and analyzed? | Were participants analyzed in the groups to which they were randomized? | Were outcomes measured in the same way for treatment groups? | Were outcomes measured in a reliable way? | Was appropriate statistical analysis used? | Was the trial design appropriate, and any deviations from the standard RCT design accounted for in the conduct and analysis of the trial. |
| --- | --- | --- | --- | --- | --- | --- | --- | --- | --- | --- | --- | --- | --- |
| Abdelalim 2021 | unclear | no | yes | no | unclear | unclear | yes | yes | yes | yes | yes | yes | yes |

Table 6. Checklist for Quasi-experimental Studies

| Study ID | Is it clear in the study what is the ‘cause’ and what is the ‘effect’ (i.e. there is no confusion about which variable comes first)? | Were the participants included in any comparisons similar? | Were the participants included in any comparisons receiving similar treatment/care, other than the exposure or intervention of interest? | Was there a control group? | Were there multiple measurements of the outcome both pre and post the intervention/exposure? | Was follow up complete and if not, were differences between groups in terms of their follow up adequately described and analyzed? | Were the outcomes of participants included in any comparisons measured in the same way? | Were outcomes measured in a reliable way? | Was appropriate statistical analysis used? | Is it clear in the study what is the ‘cause’ and what is the ‘effect’ (i.e. there is no confusion about which variable comes first)? | Were the participants included in any comparisons similar? | Were the participants included in any comparisons receiving similar treatment/care, other than the exposure or intervention of interest? |
| --- | --- | --- | --- | --- | --- | --- | --- | --- | --- | --- | --- | --- |
| LeBon 2021 | yes | yes | yes | yes | yes | yes | yes | yes | yes | yes | yes | yes |
