# Supplementary material for: Olfactory recovery following infection with COVID-19: A systematic review
Source: PLoS One. 2021 Nov 9;16(11):e0259321. doi: 10.1371/journal.pone.0259321 (PMC8577770; doi:10.1371/journal.pone.0259321)
Supplement: S2 Table — (DOCX) [file pone.0259321.s003.docx]

S2 Table. Risk/Protective factors for olfactory dysfunction and its recovery – Demographic characteristics and COVID-19 characteristics

| Study | Patient Sex | | Patient Age | | Comorbidities | | COVID-19 Severity | | COVID-19 Duration | | Development of Antibodies | |
| --- | --- | --- | --- | --- | --- | --- | --- | --- | --- | --- | --- | --- |
|  | OD Occ | OD Res | OD Occ | OD Res | OD Occ | OD Res | OD Occ | OD Res | OD Occ | OD Res | OD Occ | OD Res |
| Abdelalim 2021 | N | N |  | - |  | - (Diabetes) |  |  | + |  |  |  |
| Amer 2020 |  | + (F) | N |  |  | - (Diabetes, HTN, Allergy) |  |  |  |  |  |  |
| Biadsee 2021** |  | N |  |  |  |  |  |  |  |  |  |  |
| BrandaoNeto 2020 |  | - (F) |  |  | N |  | N |  |  |  |  |  |
| Bulgurcu 2020 |  |  |  |  |  |  | N |  |  |  |  |  |
| Chary 2020 |  | N |  | N |  |  |  | N |  |  |  |  |
| Chiesa-Estomba 2020** |  |  |  |  | N | N |  |  |  |  |  |  |
| Gonzalez 2021** |  | N |  | - |  |  |  |  |  |  |  |  |
| LeBon 2021 |  | N |  | N |  |  |  |  |  |  |  |  |
| Lechien 2020 |  | N |  | N |  |  |  |  |  |  |  |  |
| Lechien 2021 |  |  |  |  |  |  | - |  |  |  |  |  |
| Locatello 2021 |  | N |  | N |  | ___ (Allergic respiratory disease) |  |  |  |  |  |  |
| Moein 2020** | - (F) |  | + |  |  |  |  |  |  |  |  |  |
| Niklassen 2021 | N | N |  | N |  |  |  |  |  |  |  |  |
| Petrocelli 2021 |  | N |  | - (50+ years) |  |  |  |  |  |  |  |  |
| Raad 2021* |  |  |  |  | + (Asthma, history of hyposmia) |  |  |  |  |  |  |  |
| Riestra-Ayora 2021 |  |  |  |  |  |  |  |  |  |  |  | N |
| Speth 2020** | N |  |  |  |  | N |  | N |  |  |  |  |
| Ugurlu 2021 | N | - (F) | N | N |  |  |  |  |  |  |  |  |
| Vaira 2020 |  | N |  | N |  | N |  | + |  |  |  |  |
| Vaira 2020-1** |  |  |  |  |  |  |  | + |  |  |  |  |
| Vaira 2020-3** |  | N |  | N |  | N |  |  |  |  |  |  |

OD Occ= Olfactory Dysfunction Occurrence; OD Res=Olfactory Dysfunction Resolution; (-) Blue = Negative association; (+) Green = Positive association; N= No association;( ___) Orange =Unclear association; (F)= Female; HTN= Hypertension. ** Study used multivariable analyses

Table 2. Risk/Protective factors for olfactory dysfunction and its recovery –ENT Symptoms

| Study | Severity of OD | | Nasal Obstruction | | Rhinorrhea | | Facial Pain | | Olfactory cleft obstruction on MRI | | Recovery of taste | |
| --- | --- | --- | --- | --- | --- | --- | --- | --- | --- | --- | --- | --- |
|  | OD Occ | OD Res | OD Occ | OD Res | OD Occ | OD Res | OD Occ | OD Res | OD Occ | OD Res | OD Occ | OD Res |
| Bertlich 2021 |  | N |  |  |  |  |  |  |  |  |  |  |
| Biadsee 2021 |  |  |  |  |  |  |  |  |  |  |  | + |
| Boscolo-Rizzo 2020 |  |  |  | N |  |  |  |  |  |  |  |  |
| Chiesa-Estomba 2020 |  | - |  |  |  |  |  |  |  |  |  |  |
| Eliezer 2020 |  |  |  |  |  |  |  |  | + |  |  |  |
| Gorzkowski 2020 |  |  |  | - |  |  |  |  |  |  |  |  |
| Iannuzzi 2020 |  | + |  |  |  |  |  |  |  |  |  |  |
| Konstantinidis 2020 |  | - (bimodal odors |  |  |  |  |  |  |  |  |  |  |
| Lechien 2020 |  |  |  |  |  |  |  |  |  |  |  |  |
| Locatello 2021 |  |  |  | N |  | N |  |  |  |  |  |  |
| Riestra-Ayora 2021 |  |  |  | - |  |  |  |  |  |  |  |  |
| Sahoo 2021 |  |  | + |  |  |  |  |  |  |  |  |  |
| Sakalli 2020 |  |  | + |  | + |  | + |  |  |  |  |  |
| Speth 2020 |  |  |  | N |  | N |  |  |  |  |  |  |
| Speth 2020** |  | ___ |  |  |  |  |  |  |  |  |  |  |
| Ugurlu 2021 |  | - |  |  |  |  |  |  |  |  |  |  |
| Vaira 2020-3** |  | - |  |  |  |  |  |  |  |  |  |  |

OD Occ= Olfactory Dysfunction Occurrence; OD Res=Olfactory Dysfunction Resolution; (-) Blue = Negative association; (+) Green = Positive association; N= No association;( ___) Orange =Unclear association; ** Study used multivariable analyses

Table 3. Risk/Protective factors for recovery of olfactory disorder – COVID-19 symptoms

| **Study** | **Fatigue** | | **Myalgia** | | **Fever** | | **Cough** | | **Headache** | | **Dyspnea** | | **Ground glass appearance on chest CT** | | **GI disorders** | | **Median Lymphocyte count** | |
| --- | --- | --- | --- | --- | --- | --- | --- | --- | --- | --- | --- | --- | --- | --- | --- | --- | --- | --- |
|  | OD Occ | OD Res | OD Occ | OD Res | OD Occ | OD Res | OD Occ | OD Res | OD Occ | OD Res | OD Occ | OD Res | OD Occ | OD Res | OD Occ | OD Res | OD Occ | OD Res |
| Bertlich 2021 |  |  |  |  |  |  | + |  | + |  |  |  |  |  |  |  |  |  |
| Kavaz 2021 |  |  | + |  |  |  |  |  |  |  |  |  | + |  |  |  | - |  |
| Locatello 2021 |  | N |  |  |  | N |  | N |  | N |  |  |  |  |  | N |  |  |
| Petrocelli 2021 |  |  |  |  |  | N |  |  |  | N |  |  |  | N |  |  |  |  |
| Riestra-Ayora 2021 |  |  |  |  |  |  |  |  |  |  |  | - |  |  |  |  |  |  |
| Sahoo 2021 |  |  |  |  | + |  |  |  |  |  |  |  |  |  |  |  |  |  |
| Sakalli 2020 | + |  | + |  | + |  |  |  | + |  |  |  |  |  |  |  |  |  |
| Speth 2020** |  |  |  |  |  | N |  | N |  |  |  | N |  |  |  |  |  |  |
| Vaira 2020-1** |  |  |  |  |  | + |  |  |  |  |  |  |  |  |  |  |  |  |

OD Occ= Olfactory Dysfunction Occurrence; OD Res=Olfactory Dysfunction Resolution; (-) Blue = Negative association; (+) Green = Positive association; N= No association;( ___) Orange=Unclear association ; CT= computed tomography; GI= Gastro-intestinal; ** Study used multivariable analyses
