# Supplementary material for: Olfactory recovery following infection with COVID-19: A systematic review
Source: PLoS One. 2021 Nov 9;16(11):e0259321. doi: 10.1371/journal.pone.0259321 (PMC8577770; doi:10.1371/journal.pone.0259321)
Supplement: S3 Table — (DOCX) [file pone.0259321.s004.docx]

| **NCT  #** | **Title** | **Status** | **Intervention/s** |
| --- | --- | --- | --- |
| NCT04764981 | Olfactory training for olfactory dysfunction after coronavirus disease-19 (COVID-19) | Enrolling by invitation | Olfactory training with essence oils vs  No treatment |
| NCT04710394 | Visual olfactory training in participants with COVID 19 resultant loss of smell | Recruiting | Unimodal Olfactory Training with Conventional Odors  Unimodal Olfactory Training with Patient-Preferred Odors  Bimodal Visual, Olfactory Training with Conventional Odors  Bimodal Visual, Olfactory Training with Patient-Preferred Odors |
| NCT04361474 | Trial evaluating the efficacy of local Budesonide therapy in the management of hyposmia in COVID-19 patients without signs of severity | Active, not recruiting | Budesonide (nasal) vs  Physiological serum |
| NCT04657809 | Clinical assessment of insulin fast dissolving film in treatment of post infection anosmia | Active, no recruiting | Insulin fast dissolving film vs  Plain dissolving film |
| NCT04406584 | Intranasal injection of PRP versus saline for treatment of olfactory dysfunction | Not yet recruiting | Platelet-rich plasma injection into olfactory cleft vs  Saline |
| NCT04495816 | COVID-19 Anosmia study | Recruiting | Omega-3 Fatty acid supplement vs  Placebo |
| NCT04569825 | Effect of nasal steroid in the treatment of anosmia due to COVID-19 disease | Recruiting | Ophtamesone vs  Normal Saline |
| NCT04789499 | Smell in COVID 19 and efficacy of Nasal Theophylline | Recruiting | Theophylline Nasal irrigation vs  Placebo |
| NCT04528329 | Time to recover of anosmia and/or ageusia and early corticosteroid use | Recruiting | Corticosteroid (Dexamethasone) |

S3 Table. Ongoing studies Evaluating Treatments for Olfactory Dysfunction due to COVID-19 Infection
