# Supplementary material for: Olfactory recovery following infection with COVID-19: A systematic review
Source: PLoS One. 2021 Nov 9;16(11):e0259321. doi: 10.1371/journal.pone.0259321 (PMC8577770; doi:10.1371/journal.pone.0259321)
Supplement: S1 Text — (DOCX) [file pone.0259321.s006.docx]

Database: Embase Classic+Embase <1947 to 2020 December 17> , Ovid MEDLINE(R) ALL <1946 to December 17, 2020>, EBM Reviews - Cochrane Central Register of Controlled Trials <November 2020>

Search Strategy:

--------------------------------------------------------------------------------

1 (exp coronavirus/ or coronavirus*.mp. or corona virus*.mp.) and (wuhan or beijing or shanghai or hubei).mp. (9021)

2 ((coronavirus* or corona virus* or coronavirus* or coronaviridae or coronaviridae or betacoronavirus*) adj3 ("19" or "2019")).tw. (44956)

3 covid.tw,kw. (149166)

4 covid19.tw,kw. or covid 19.kw. (63187)

5 sars cov 2.tw,kw. (52681)

6 (ncov or n cov).tw,kw. (2850)

7 (novel coronavirus* or novel corona virus*).tw,kw. (13689)

8 (CoV 2 or CoV2 or sarscov2 or 2019nCoV or novel CoV or wuhan virus*).tw,kw. (50845)

9 (Coronavirus Infections/ or Severe Acute Respiratory Syndrome/) and (Pandemics/ or pandemic*.tw,kf.) (43575)

10 or/1-9 (170245)

11 Olfaction Disorders/ or smell/ or Sensation Disorders/ (69407)

12 smell*.tw,kw. (28726)

13 olfact*.tw,kw. (118425)

14 anosmi*.tw,kw. (7986)

15 (dysosmi* or Hyposmi*).tw,kw. (4725)

16 or/11-15 (177589)

17 10 and 16 (2048)

18 exp animals/ not humans/ (18522022)

19 17 not 18 (1101)

**20 19 use medall (975) Medline**

21 (2020072* or 2020073* or 202008* or 202009* or 20201*).dt. (661432)

**22 20 and 21 (572) Medline Update**

23 (Coronavirinae/ or coronavirus*.mp. or corona virus*.mp.) and (wuhan or beijing or shanghai or hubei).mp. (8992)

24 ((coronavirus* or corona virus* or coronavirus* or coronaviridae or coronaviridae or betacoronavirus*) adj3 ("19" or "2019")).tw. (44956)

25 (covid or covid19).tw. (147209)

26 sars cov 2.tw. (45051)

27 (ncov or n cov).tw. (2494)

28 (novel coronavirus* or novel corona virus*).tw. (12858)

29 (CoV 2 or CoV2 or sarscov2 or 2019nCoV or novel CoV or wuhan virus).tw. (46847)

30 (coronavirus infection/ or severe acute respiratory syndrome/) and (pandemic/ or pandemic*.tw.) (51284)

31 limit 30 to yr="2019 -Current" (50184)

32 23 or 24 or 25 or 26 or 27 or 28 or 29 or 31 (168246)

33 exp smelling disorder/ (12313)

34 (smell* or olfact*).tw. (133919)

35 (anosmi* or dysosmi* or Hyposmi*).tw. (10440)

36 or/33-35 (142112)

37 32 and 36 (2352)

38 (exp animal/ or nonhuman/) not exp human/ (12074496)

39 37 not 38 (2344)

**40 39 use emczd (1352) Embase**

41 (2020072* or 2020073* or 202008* or 202009* or 20201*).dc. (1123677)

**42 40 and 41 (1113) Embase update**

43 (exp coronavirus/ or coronavirus*.mp. or corona virus*.mp.) and (wuhan or beijing or shanghai or hubei).mp. (9021)

44 ((coronavirus* or corona virus* or coronavirus* or coronaviridae or coronaviridae or betacoronavirus*) adj3 ("19" or "2019")).tw. (44956)

45 covid.tw,kw. (149166)

46 covid19.tw,kw. or covid 19.kw. (63187)

47 sars cov 2.tw,kw. (52681)

48 (ncov or n cov).tw,kw. (2850)

49 (novel coronavirus* or novel corona virus*).tw,kw. (13689)

50 (CoV 2 or CoV2 or sarscov2 or 2019nCoV or novel CoV or wuhan virus*).tw,kw. (50845)

51 (Coronavirus Infections/ or Severe Acute Respiratory Syndrome/) and (Pandemics/ or pandemic*.tw.) (43570)

52 or/43-51 (170244)

53 Olfaction Disorders/ or smell/ or Sensation Disorders/ (69407)

54 smell*.tw,kw. (28726)

55 olfact*.tw,kw. (118425)

56 anosmi*.tw,kw. (7986)

57 (dysosmi* or Hyposmi*).tw,kw. (4725)

58 or/53-57 (177589)

59 52 and 58 (2048)

**60 59 use cctr (71) Cochrane**

61 limit 60 to yr="2020 -Current" (71)

62 22 or 42 or 61 (1756)

63 remove duplicates from 62 (1282)

**64 63 use medall (557)**

**65 63 use emczd (656)**

**66 63 use cctr (69)**
