# Supplementary material for: Olfactory recovery following infection with COVID-19: A systematic review
Source: PLoS One. 2021 Nov 9;16(11):e0259321. doi: 10.1371/journal.pone.0259321 (PMC8577770; doi:10.1371/journal.pone.0259321)
Supplement: S2 Text — (DOCX) [file pone.0259321.s007.docx]

**Wrong study design**

1. Alvarado GR, Pierson BC, Teemer ES, Gama HJ, Cole RD, Jang SS. Symptom Characterization and Outcomes of Sailors in Isolation After a COVID-19 Outbreak on a US Aircraft Carrier. JAMA Netw Open. 2020;3(10):e2020981.

2. Aragão M, Leal MC, Cartaxo Filho OQ, Fonseca TM, Valença MM. Anosmia in COVID-19 Associated with Injury to the Olfactory Bulbs Evident on MRI. AJNR Am J Neuroradiol. 2020;41(9):1703-6.

3. Brelie LF, Becker C, Brelie CV. Parosmia as an Early Symptom of Acute SARS-CoV-2 Infection. Dtsch Arztebl Int. 2020;117(18):328.

4. Bulbuloglu S, Altun Y. The effect of sniffing Turkish coffee on olfactory disorders in COVID-19 patients: An experimental clinical study. Ideggyogy Sz. 2021;74(3-4):117-23.

5. Chen C, Chen M, Cheng C, Chi Y, Hu Z, Liu Y, et al. A special symptom of olfactory dysfunction in coronavirus disease 2019: report of three cases. J Neurovirol. 2020;26(3):456-8.

6. Chua AJ, Charn TC, Chan EC, Loh J. Acute Olfactory Loss Is Specific for COVID-19 at the Emergency Department. Ann Emerg Med. 2020;76(4):550-1.

7. Dawson P, Rabold EM, Laws RL, Conners EE, Gharpure R, Yin S, et al. Loss of Taste and Smell as Distinguishing Symptoms of Coronavirus Disease 2019. Clin Infect Dis. 2021;72(4):682-5.

8. Eliezer M, Hautefort C, Hamel AL, Verillaud B, Herman P, Houdart E, et al. Sudden and Complete Olfactory Loss of Function as a Possible Symptom of COVID-19. JAMA Otolaryngol Head Neck Surg. 2020;146(7):674-5.

9. Fjaeldstad AW. Prolonged complaints of chemosensory loss after COVID-19. Dan Med J. 2020;67(8).

10. Gaborieau L, Delestrain C, Bensaid P, Vizeneux A, Blanc P, Garraffo A, et al. Epidemiology and Clinical Presentation of Children Hospitalized with SARS-CoV-2 Infection in Suburbs of Paris. J Clin Med. 2020;9(7).

11. Gane SB, Kelly C, Hopkins C. Isolated sudden onset anosmia in COVID-19 infection. A novel syndrome? Rhinology. 2020;58(3):299-301.

12. Giacomelli A, Pezzati L, Conti F, Bernacchia D, Siano M, Oreni L, et al. Self-reported Olfactory and Taste Disorders in Patients With Severe Acute Respiratory Coronavirus 2 Infection: A Cross-sectional Study. Clin Infect Dis. 712020. p. 889-90.

13. Gilani S, Roditi R, Naraghi M. COVID-19 and anosmia in Tehran, Iran. Med Hypotheses. 2020;141:109757.

14. Gómez-Iglesias P, Porta-Etessam J, Montalvo T, Valls-Carbó A, Gajate V, Matías-Guiu JA, et al. An Online Observational Study of Patients With Olfactory and Gustory Alterations Secondary to SARS-CoV-2 Infection. Front Public Health. 2020;8:243.

15. Hua-Huy T, Lorut C, Aubourg F, Morbieu C, Marey J, Texereau J, et al. Persistent Nasal Inflammation 5 Months after Acute Anosmia in Patients with COVID-19. Am J Respir Crit Care Med. 2021;203(10):1319-22.

16. Iravani B, Arshamian A, Ravia A, Mishor E, Snitz K, Shushan S, et al. Relationship between odor intensity estimates and COVID-19 prevalence prediction in a Swedish population. Chem Senses. 2020.

17. Izquierdo-Domínguez A, Rojas-Lechuga MJ, Mullol J, Alobid I. Olfactory dysfunction during COVID-19 pandemic. Med Clin (Engl Ed). 2020;155(9):403-8.

18. Kaye R, Chang CWD, Kazahaya K, Brereton J, Denneny JC, 3rd. COVID-19 Anosmia Reporting Tool: Initial Findings. Otolaryngol Head Neck Surg. 2020;163(1):132-4.

19. Klopfenstein T, Kadiane-Oussou NJ, Toko L, Royer PY, Lepiller Q, Gendrin V, et al. Features of anosmia in COVID-19. Med Mal Infect. 2020;50(5):436-9.

20. Kumar V, Singla S, Gupta N, Bharati SJ, Garg R, Pandit A, et al. The incidence of anosmia in patients with laboratory-confirmed COVID 19 infection in India: An observational study. J Anaesthesiol Clin Pharmacol. 2021;37(1):51-6.

21. Lao WP, Imam SA, Nguyen SA. Anosmia, hyposmia, and dysgeusia as indicators for positive SARS-CoV-2 infection. World J Otorhinolaryngol Head Neck Surg. 2020;6(Suppl 1):S22-s5.

22. Lechien JR, Cabaraux P, Chiesa-Estomba CM, Khalife M, Hans S, Calvo-Henriquez C, et al. Objective olfactory evaluation of self-reported loss of smell in a case series of 86 COVID-19 patients. Head Neck. 2020;42(7):1583-90.

23. Lechien JR, Cabaraux P, Chiesa-Estomba CM, Khalife M, Plzak J, Hans S, et al. Psychophysical Olfactory Tests and Detection of COVID-19 in Patients With Sudden Onset Olfactory Dysfunction: A Prospective Study. Ear Nose Throat J. 2020;99(9):579-83.

24. Lechien JR, Chiesa-Estomba CM, Hans S, Barillari MR, Jouffe L, Saussez S. Loss of Smell and Taste in 2013 European Patients With Mild to Moderate COVID-19. Ann Intern Med. 1732020. p. 672-5.

25. Lechien JR, Chiesa-Estomba CM, Place S, Van Laethem Y, Cabaraux P, Mat Q, et al. Clinical and epidemiological characteristics of 1420 European patients with mild-to-moderate coronavirus disease 2019. J Intern Med. 2020;288(3):335-44.

26. Lee DJ, Lockwood J, Das P, Wang R, Grinspun E, Lee JM. Self-reported anosmia and dysgeusia as key symptoms of coronavirus disease 2019. Cjem. 2020;22(5):595-602.

27. Levy JM. Treatment Recommendations for Persistent Smell and Taste Dysfunction Following COVID-19-The Coming Deluge. JAMA Otolaryngol Head Neck Surg. 2020;146(8):733.

28. Li J, Long X, Zhu C, Wang H, Wang T, Lin Z, et al. Olfactory Dysfunction in Recovered Coronavirus Disease 2019 (COVID-19) Patients. Mov Disord. 352020. p. 1100-1.

29. Liang Y, Xu J, Chu M, Mai J, Lai N, Tang W, et al. Neurosensory dysfunction: A diagnostic marker of early COVID-19. Int J Infect Dis. 2020;98:347-52.

30. Liu PY, Jiang RS. Prognosis of olfactory and gustatory dysfunctions in COVID-19 patients: A case series. Clin Case Rep. 8: © 2020 The Authors. Clinical Case Reports published by John Wiley & Sons Ltd.; 2020. p. 2744-52.

31. Lombardi A, Consonni D, Carugno M, Bozzi G, Mangioni D, Muscatello A, et al. Characteristics of 1573 healthcare workers who underwent nasopharyngeal swab testing for SARS-CoV-2 in Milan, Lombardy, Italy. Clin Microbiol Infect. 2020;26(10):1413.e9-.e13.

32. Lovato A, Galletti C, Galletti B, de Filippis C. Clinical characteristics associated with persistent olfactory and taste alterations in COVID-19: A preliminary report on 121 patients. Am J Otolaryngol. 2020;41(5):102548.

33. Luers JC, Rokohl AC, Loreck N, Wawer Matos PA, Augustin M, Dewald F, et al. Olfactory and Gustatory Dysfunction in Coronavirus Disease 2019 (COVID-19). Clin Infect Dis. 2020;71(16):2262-4.

34. Macera M, De Angelis G, Sagnelli C, Coppola N, Vanvitelli C-G. Clinical Presentation of COVID-19: Case Series and Review of the Literature. Int J Environ Res Public Health. 2020;17(14).

35. Mak PQ, Chung KS, Wong JS, Shek CC, Kwan MY. Anosmia and Ageusia: Not an Uncommon Presentation of COVID-19 Infection in Children and Adolescents. Pediatr Infect Dis J. 2020;39(8):e199-e200.

36. Meini S, Suardi LR, Busoni M, Roberts AT, Fortini A. Olfactory and gustatory dysfunctions in 100 patients hospitalized for COVID-19: sex differences and recovery time in real-life. Eur Arch Otorhinolaryngol. 2020;277(12):3519-23.

37. Mendonça CV, Mendes Neto JA, Suzuki FA, Orth MS, Machado Neto H, Nacif SR. Olfactory dysfunction in COVID-19: a marker of good prognosis? Braz J Otorhinolaryngol. 2021.

38. Menni C, Valdes AM, Freidin MB, Sudre CH, Nguyen LH, Drew DA, et al. Real-time tracking of self-reported symptoms to predict potential COVID-19. Nat Med. 2020;26(7):1037-40.

39. Mermelstein S. Acute anosmia from COVID-19 infection. Pract Neurol. 2020;20(4):343-4.

40. Merza MA, Haleem Al Mezori AA, Mohammed HM, Abdulah DM. COVID-19 outbreak in Iraqi Kurdistan: The first report characterizing epidemiological, clinical, laboratory, and radiological findings of the disease. Diabetes Metab Syndr. 2020;14(4):547-54.

41. Mimenza-Alvarado AJ, Avila-Funes JA, Aguilar-Navarro SG. OLFACTORY DISORDERS IN SARS-COV-2 INFECTION: CONSIDERATIONS IN AGING. Rev Invest Clin. 2020;72(3):135-7.

42. Moein ST, Hashemian SM, Mansourafshar B, Khorram-Tousi A, Tabarsi P, Doty RL. Smell dysfunction: a biomarker for COVID-19. Int Forum Allergy Rhinol. 2020;10(8):944-50.

43. Naeini AS, Karimi-Galougahi M, Raad N, Ghorbani J, Taraghi A, Haseli S, et al. Paranasal sinuses computed tomography findings in anosmia of COVID-19. Am J Otolaryngol. 2020;41(6):102636.

44. Paderno A, Schreiber A, Grammatica A, Raffetti E, Tomasoni M, Gualtieri T, et al. Smell and taste alterations in COVID-19: a cross-sectional analysis of different cohorts. Int Forum Allergy Rhinol. 2020;10(8):955-62.

45. Paolo G. Does COVID-19 cause permanent damage to olfactory and gustatory function? Med Hypotheses. 2020;143:110086.

46. Patel A, Charani E, Ariyanayagam D, Abdulaal A, Denny SJ, Mughal N, et al. New-onset anosmia and ageusia in adult patients diagnosed with SARS-CoV-2 infection. Clin Microbiol Infect. 2020;26(9):1236-41.

47. Rebholz H, Pfaffeneder-Mantai F, Knoll W, Hassel AW, Frank W, Kleber C. Olfactory dysfunction in SARS-CoV-2 infection: Focus on odorant specificity and chronic persistence. Am J Otolaryngol. 2021;42(5):103014.

48. Roland LT, Gurrola JG, 2nd, Loftus PA, Cheung SW, Chang JL. Smell and taste symptom-based predictive model for COVID-19 diagnosis. Int Forum Allergy Rhinol. 2020;10(7):832-8.

49. Sayin İ, Yaşar KK, Yazici ZM. Taste and Smell Impairment in COVID-19: An AAO-HNS Anosmia Reporting Tool-Based Comparative Study. Otolaryngol Head Neck Surg. 2020;163(3):473-9.

50. Sbrana MF, Fornazieri MA, Bruni-Cardoso A, Avelino-Silva VI, Schechtman D, Voegels RL, et al. Olfactory Dysfunction in Frontline Health Care Professionals During COVID-19 Pandemic in Brazil. Front Physiol. 2021;12:622987.

51. Schmithausen RM, Döhla M, Schöβler H, Diegmann C, Schulte B, Richter E, et al. Characteristic Temporary Loss of Taste and Olfactory Senses in SARS-CoV-2-positive-Individuals with Mild Symptoms. Pathog Immun. 2020;5(1):117-20.

52. Shah NN, Hussain RT, Mustafa H, Mushtaq M, Ali M. Evaluation of Olfactory Acuity in Patients with Coronavirus Disease 2019 (COVID-19). Indian J Otolaryngol Head Neck Surg. 2020:1-8.

53. Sierpiński R, Pinkas J, Jankowski M, Zgliczyński WS, Wierzba W, Gujski M, et al. Sex differences in the frequency of gastrointestinal symptoms and olfactory or taste disorders in 1942 nonhospitalized patients with coronavirus disease 2019 (COVID-19). Pol Arch Intern Med. 2020;130(6):501-5.

54. Speth MM, Singer-Cornelius T, Oberle M, Gengler I, Brockmeier SJ, Sedaghat AR. Olfactory Dysfunction and Sinonasal Symptomatology in COVID-19: Prevalence, Severity, Timing, and Associated Characteristics. Otolaryngol Head Neck Surg. 2020;163(1):114-20.

55. Tomlins J, Hamilton F, Gunning S, Sheehy C, Moran E, MacGowan A. Clinical features of 95 sequential hospitalised patients with novel coronavirus 2019 disease (COVID-19), the first UK cohort. J Infect. 2020;81(2):e59-e61.

56. Tsivgoulis G, Fragkou PC, Delides A, Karofylakis E, Dimopoulou D, Sfikakis PP, et al. Quantitative evaluation of olfactory dysfunction in hospitalized patients with Coronavirus [2] (COVID-19). J Neurol. 2672020. p. 2193-5.

57. Tudrej B, Sebo P, Lourdaux J, Cuzin C, Floquet M, Haller DM, et al. Self-Reported Loss of Smell and Taste in SARS-CoV-2 Patients: Primary Care Data to Guide Future Early Detection Strategies. J Gen Intern Med. 352020. p. 2502-4.

58. Vaira LA, Deiana G, Fois AG, Pirina P, Madeddu G, De Vito A, et al. Objective evaluation of anosmia and ageusia in COVID-19 patients: Single-center experience on 72 cases. Head Neck. 2020;42(6):1252-8.

59. Vaira LA, Salzano G, Petrocelli M, Deiana G, Salzano FA, De Riu G. Validation of a self-administered olfactory and gustatory test for the remotely evaluation of COVID-19 patients in home quarantine. Head Neck. 2020;42(7):1570-6.

60. Walker A, Pottinger G, Scott A, Hopkins C. Anosmia and loss of smell in the era of covid-19. Bmj. 2020;370:m2808.

61. Wee LE, Chan YFZ, Teo NWY, Cherng BPZ, Thien SY, Wong HM, et al. The role of self-reported olfactory and gustatory dysfunction as a screening criterion for suspected COVID-19. Eur Arch Otorhinolaryngol. 2772020. p. 2389-90.

62. Whitcroft KL, Hummel T. Olfactory Dysfunction in COVID-19: Diagnosis and Management. Jama. 2020;323(24):2512-4.

63. Yan CH, Faraji F, Prajapati DP, Ostrander BT, DeConde AS. Self-reported olfactory loss associates with outpatient clinical course in COVID-19. Int Forum Allergy Rhinol. 2020;10(7):821-31.

64. Zayet S, Klopfenstein T, Mercier J, Kadiane-Oussou NJ, Lan Cheong Wah L, Royer PY, et al. Contribution of anosmia and dysgeusia for diagnostic of COVID-19 in outpatients. Infection. 492021. p. 361-5.

**No follow up data**

1. Abalo-Lojo JM, Pouso-Diz JM, Gonzalez F. Taste and Smell Dysfunction in COVID-19 Patients. Ann Otol Rhinol Laryngol. 2020;129(10):1041-2.

2. Altin F, Cingi C, Uzun T, Bal C. Olfactory and gustatory abnormalities in COVID-19 cases. Eur Arch Otorhinolaryngol. 2020;277(10):2775-81.

3. Altundag A, Saatci O, Sanli DET, Duz OA, Sanli AN, Olmuscelik O, et al. The temporal course of COVID-19 anosmia and relation to other clinical symptoms. Eur Arch Otorhinolaryngol. 2021;278(6):1891-7.

4. Antolín-Amérigo D, Cubero JL, Colás C, Alobid I, Mullol J, Valero A. High frequency of smell and taste dysfunctions in allergy health care professionals suffering from COVID-19. J Investig Allergol Clin Immunol. 2021:0.

5. Biadsee A, Kassem F, Dagan O, Masarwa S, Ormianer Z. Olfactory and Oral Manifestations of COVID-19: Sex-Related Symptoms-A Potential Pathway to Early Diagnosis. Otolaryngol Head Neck Surg. 2020;163(4):722-8.

6. Carignan A, Valiquette L, Grenier C, Musonera JB, Nkengurutse D, Marcil-Héguy A, et al. Anosmia and dysgeusia associated with SARS-CoV-2 infection: an age-matched case-control study. Cmaj. 2020;192(26):E702-e7.

7. Chiesa-Estomba CM, Lechien JR, Portillo-Mazal P, Martínez F, Cuauro-Sanchez J, Calvo-Henriquez C, et al. Olfactory and gustatory dysfunctions in COVID-19. First reports of Latin-American ethnic patients. Am J Otolaryngol. 2020;41(5):102605.

8. Cho RHW, To ZWH, Yeung ZWC, Tso EYK, Fung KSC, Chau SKY, et al. COVID-19 Viral Load in the Severity of and Recovery From Olfactory and Gustatory Dysfunction. Laryngoscope. 2020;130(11):2680-5.

9. Chung TW, Sridhar S, Zhang AJ, Chan KH, Li HL, Wong FK, et al. Olfactory Dysfunction in Coronavirus Disease 2019 Patients: Observational Cohort Study and Systematic Review. Open Forum Infect Dis. 2020;7(6):ofaa199.

10. Dağlı HY, Akcan ÖM, Pekcan S, Gençeli M, Özdemir HS, Uğurlu B, et al. Uncommon Presentation of COVID-19 in Pediatric Patients: Anosmia. Journal of Pediatric Infectious Diseases. 2021;16(02):080-4.

11. Gerkin RC, Ohla K, Veldhuizen MG, Joseph PV, Kelly CE, Bakke AJ, et al. Recent smell loss is the best predictor of COVID-19: a preregistered, cross-sectional study. medRxiv. 2020.

12. Gözen ED, Aliyeva C, Tevetoğlu F, Karaali R, Balkan İ, Yener HM, et al. Evaluation of Olfactory Function With Objective Tests in COVID-19-Positive Patients: A Cross-Sectional Study. Ear Nose Throat J. 2021;100(2_suppl):169s-73s.

13. Gupta V, Banavara Rajanna L, Upadhyay K, Bhatia R, Madhav Reddy N, Malik D, et al. Olfactory and Gustatory Dysfunction in COVID-19 Patients from Northern India: A Cross-Sectional Observational Study. Indian J Otolaryngol Head Neck Surg. 2021;73(2):1-8.

14. Haehner A, Draf J, Dräger S, de With K, Hummel T. Predictive Value of Sudden Olfactory Loss in the Diagnosis of COVID-19. ORL J Otorhinolaryngol Relat Spec. 2020;82(4):175-80.

15. Hintschich CA, Wenzel JJ, Hummel T, Hankir MK, Kühnel T, Vielsmeier V, et al. Psychophysical tests reveal impaired olfaction but preserved gustation in COVID-19 patients. Int Forum Allergy Rhinol. 102020. p. 1105-7.

16. Hornuss D, Lange B, Schröter N, Rieg S, Kern WV, Wagner D. Anosmia in COVID-19 patients. Clin Microbiol Infect. 2020;26(10):1426-7.

17. İşlek A, Balcı MK. Evaluation of effects of chronic nasal steroid use on rhinological symptoms of COVID-19 with SNOT-22 questionnaire. Pharmacol Rep. 2021;73(3):781-5.

18. Izquierdo-Domínguez A, Rojas-Lechuga MJ, Chiesa-Estomba C, Calvo-Henríquez C, Ninchritz-Becerra E, Soriano-Reixach M, et al. Smell and Taste Dysfunction in COVID-19 Is Associated With Younger Age in Ambulatory Settings: A Multicenter Cross-Sectional Study. J Investig Allergol Clin Immunol. 2020;30(5):346-57.

19. Kandakure VT, Valvi HR, Khokle P, More MS, Chouhan R. Prevalence and Recovery from Newly Onset Anosmia and Ageusia in Covid 19 Patients at our Teritary Care Centre. Indian J Otolaryngol Head Neck Surg. 2021:1-8.

20. Klein H, Asseo K, Karni N, Benjamini Y, Nir-Paz R, Muszkat M, et al. Onset, duration and unresolved symptoms, including smell and taste changes, in mild COVID-19 infection: a cohort study in Israeli patients. Clin Microbiol Infect. 2021;27(5):769-74.

21. Le Bon SD, Pisarski N, Verbeke J, Prunier L, Cavelier G, Thill MP, et al. Psychophysical evaluation of chemosensory functions 5 weeks after olfactory loss due to COVID-19: a prospective cohort study on 72 patients. Eur Arch Otorhinolaryngol. 2021;278(1):101-8.

22. Lechien JR, Chiesa-Estomba CM, De Siati DR, Horoi M, Le Bon SD, Rodriguez A, et al. Olfactory and gustatory dysfunctions as a clinical presentation of mild-to-moderate forms of the coronavirus disease (COVID-19): a multicenter European study. Eur Arch Otorhinolaryngol. 2020;277(8):2251-61.

23. Lechien JR, Chiesa-Estomba CM, Vaira LA, De Riu G, Cammaroto G, Chekkoury-Idrissi Y, et al. Epidemiological, otolaryngological, olfactory and gustatory outcomes according to the severity of COVID-19: a study of 2579 patients. Eur Arch Otorhinolaryngol. 2021;278(8):2851-9.

24. Lechien JR, Ducarme M, Place S, Chiesa-Estomba CM, Khalife M, De Riu G, et al. Objective Olfactory Findings in Hospitalized Severe COVID-19 Patients. Pathogens. 2020;9(8).

25. Lechien JR, Michel J, Radulesco T, Chiesa-Estomba CM, Vaira LA, De Riu G, et al. Clinical and Radiological Evaluations of COVID-19 Patients With Anosmia: Preliminary Report. Laryngoscope. 2020;130(11):2526-31.

26. Lechner M, Chandrasekharan D, Jumani K, Liu J, Gane S, Lund VJ, et al. Anosmia as a presenting symptom of SARS-CoV-2 infection in healthcare workers - A systematic review of the literature, case series, and recommendations for clinical assessment and management. Rhinology. 2020;58(4):394-9.

27. Lechner M, Liu J, Counsell N, Ta NH, Rocke J, Anmolsingh R, et al. Course of symptoms for loss of sense of smell and taste over time in one thousand forty-one healthcare workers during the Covid-19 pandemic: Our experience. Clin Otolaryngol. 462021. p. 451-7.

28. Mangia LRL, Soares MB, de Souza TSC, De Masi RDJ, Scarabotto PC, Hamerschmidt R. Objective evaluation and predictive value of olfactory dysfunction among patients hospitalized with COVID-19. Auris Nasus Larynx. 2021;48(4):770-6.

29. Martin-Sanz E, Riestra J, Yebra L, Larran A, Mancino F, Yanes-Diaz J, et al. Prospective Study in 355 Patients With Suspected COVID-19 Infection: Value of Cough, Subjective Hyposmia, and Hypogeusia. Laryngoscope. 2020;130(11):2674-9.

30. Milone I, Vento R, Ippolito L, Paroni S, Vento MG. Therapeutic support protocol for patient with dysosmia with or without dysgeusia related to the SARS-CoV2 virus infection. Acta Biomed. 2020;91(4):e2020130.

31. Ninchritz-Becerra E, Soriano-Reixach MM, Mayo-Yánez M, Calvo-Henríquez C, Martínez-Ruiz de Apodaca P, Saga-Gutiérrez C, et al. Subjective evaluation of smell and taste dysfunction in patients with mild COVID-19 in Spain. Med Clin (Engl Ed). 156: © 2020 Elsevier España, S.L.U. All rights reserved.; 2021. p. 61-4.

32. Özçelik Korkmaz M, Eğilmez OK, Özçelik MA, Güven M. Otolaryngological manifestations of hospitalised patients with confirmed COVID-19 infection. Eur Arch Otorhinolaryngol. 2021;278(5):1675-85.

33. Petrocelli M, Ruggiero F, Baietti AM, Pandolfi P, Salzano G, Salzano FA, et al. Remote psychophysical evaluation of olfactory and gustatory functions in early-stage coronavirus disease 2019 patients: the Bologna experience of 300 cases. J Laryngol Otol. 2020;134(7):571-6.

34. Qiu C, Cui C, Hautefort C, Haehner A, Zhao J, Yao Q, et al. Olfactory and Gustatory Dysfunction as An Early Identifier of COVID-19 in Adults and Children: An International Multicenter Study. medRxiv. 2020.

35. Ramasamy K, Saniasiaya J, Abdul Gani N. Olfactory and Gustatory Dysfunctions as a Clinical Manifestation of Coronavirus Disease 2019 in a Malaysian Tertiary Center. Ann Otol Rhinol Laryngol. 2021;130(5):513-9.

36. Rass V, Beer R, Schiefecker AJ, Kofler M, Lindner A, Mahlknecht P, et al. Neurological outcome and quality of life 3 months after COVID-19: A prospective observational cohort study. Eur J Neurol. 2021.

37. Seden N, Yiğit E, Yiğit Ö, Kaygısız İ. Objective evaluation of odor loss in COVID-19 and other suspected cases. Am J Otolaryngol. 2021;42(1):102761.

38. Seo MY, Seok H, Hwang SJ, Choi HK, Jeon JH, Sohn JW, et al. Trend of Olfactory and Gustatory Dysfunction in COVID-19 Patients in a Quarantine Facility. J Korean Med Sci. 2020;35(41):e375.

39. Tarifi A, Al Shdaifat AA, Al-Shudifat AM, Azab M, Ismail J, Bashir R, et al. Clinical, sinonasal, and long-term smell and taste outcomes in mildly symptomatic COVID-19 patients. Int J Clin Pract. 2021;75(7):e14260.

40. Villarreal IM, Morato M, Martínez-RuizCoello M, Navarro A, Garcia-Chillerón R, Ruiz Á, et al. Olfactory and taste disorders in healthcare workers with COVID-19 infection. Eur Arch Otorhinolaryngol. 2021;278(6):2123-7.

**Wrong outcomes**

1. Abdelmaksoud AA, Ghweil AA, Hassan MH, Rashad A, Khodeary A, Aref ZF, et al. Olfactory Disturbances as Presenting Manifestation Among Egyptian Patients with COVID-19: Possible Role of Zinc. Biol Trace Elem Res. 2021:1-8.

2. Amanat M, Rezaei N, Roozbeh M, Shojaei M, Tafakhori A, Zoghi A, et al. Neurological manifestations as the predictors of severity and mortality in hospitalized individuals with COVID-19: a multicenter prospective clinical study. BMC Neurol. 2021;21(1):116.

3. Andrews PJ, Pendolino AL, Ottaviano G, Scarpa B, Grant J, Gaudioso P, et al. Olfactory and taste dysfunction among mild-to-moderate symptomatic COVID-19 positive health care workers: An international survey. Laryngoscope Investig Otolaryngol. 2020;5(6):1019-28.

4. Armange L, Bénézit F, Picard L, Pronier C, Guillot S, Lentz PA, et al. Prevalence and characteristics of persistent symptoms after non-severe COVID-19: a prospective cohort study. Eur J Clin Microbiol Infect Dis. 2021:1-5.

5. Capelli M, Gatti P. Anosmia in the first coronavirus disease 2019 outbreak in Europe: functional recovery after eight months. J Laryngol Otol. 2021;135(3):224-8.

6. Cazzolla AP, Lovero R, Lo Muzio L, Testa NF, Schirinzi A, Palmieri G, et al. Taste and Smell Disorders in COVID-19 Patients: Role of Interleukin-6. ACS Chem Neurosci. 2020;11(17):2774-81.

7. Cousyn L, Sellem B, Palich R, Bendetowicz D, Agher R, Delorme C, et al. Olfactory and gustatory dysfunctions in COVID-19 outpatients: A prospective cohort study. Infect Dis Now. 2021;51(5):440-4.

8. D'Ascanio L, Pandolfini M, Cingolani C, Latini G, Gradoni P, Capalbo M, et al. Olfactory Dysfunction in COVID-19 Patients: Prevalence and Prognosis for Recovering Sense of Smell. Otolaryngol Head Neck Surg. 2021;164(1):82-6.

9. Díaz-Reyna D, Pineda-Cásares F, Andrade-Galicia A, Aguilar-García CR, Gutiérrez-Ortiz M, Gelover-Manzo R. Frecuencia de anosmia y disgeusia en pacientes hospitalizados con SARS-CoV-2. Med Int Méx [Internet]. 2021; 3**7**(1):[56-61 pp.].

10. Hopkins C, Surda P, Vaira LA, Lechien JR, Safarian M, Saussez S, et al. Six month follow-up of self-reported loss of smell during the COVID-19 pandemic. Rhinology. 2021;59(1):26-31.

11. Horvath L, Lim JWJ, Taylor JW, Saief T, Stuart R, Rimmer J, et al. Smell and taste loss in COVID-19 patients: assessment outcomes in a Victorian population. Acta Otolaryngol. 2021;141(3):299-302.

12. Jalessi M, Bagheri SH, Azad Z, Firouzabadi FD, Amini E, Alizadeh R, et al. The outcome of olfactory impairment in patients with otherwise paucisymptomatic coronavirus disease 2019 during the pandemic. J Laryngol Otol. 2021;135(5):426-35.

13. Joffily L, Ungierowicz A, David AG, Melo B, Brito CLT, Mello L, et al. The close relationship between sudden loss of smell and COVID-19. Braz J Otorhinolaryngol. 2020;86(5):632-8.

14. Karimi-Galougahi M, Safavi Naini A, Ghorbani J, Raad N, Raygani N. Emergence and Evolution of Olfactory and Gustatory Symptoms in Patients with COVID-19 in the Outpatient Setting. Indian J Otolaryngol Head Neck Surg. 2020:1-7.

15. Karthikeyan P, Sivanand N, Vijayan N, Latheef MN. A Clinical Study of Smell Disorders in COVID-19 Patients in a Tertiary Care Hospital in Pondicherry: A Cross Sectional Study. Indian J Otolaryngol Head Neck Surg. 2021:1-6.

16. Kosugi EM, Lavinsky J, Romano FR, Fornazieri MA, Luz-Matsumoto GR, Lessa MM, et al. Incomplete and late recovery of sudden olfactory dysfunction in COVID-19. Braz J Otorhinolaryngol. 2020;86(4):490-6.

17. Lee DJ, Daliyot D, Wang R, Lockwood J, Das P, Zimlichman E, et al. Comparative Study of Chemosensory Dysfunction in COVID-19 in 2 Geographically Distinct Regions. Ear Nose Throat J. 2021:1455613211000170.

18. Lee Y, Min P, Lee S, Kim SW. Prevalence and Duration of Acute Loss of Smell or Taste in COVID-19 Patients. J Korean Med Sci. 2020;35(18):e174.

19. Levinson R, Elbaz M, Ben-Ami R, Shasha D, Levinson T, Choshen G, et al. Time course of anosmia and dysgeusia in patients with mild SARS-CoV-2 infection. Infect Dis (Lond). 2020;52(8):600-2.

20. Loftus PA, Roland LT, Gurrola JG, 2nd, Cheung SW, Chang JL. Temporal Profile of Olfactory Dysfunction in COVID-19. OTO Open. 2020;4(4):2473974x20978133.

21. Lucidi D, Molinari G, Silvestri M, De Corso E, Guaraldi G, Mussini C, et al. Patient-reported olfactory recovery after SARS-CoV-2 infection: A 6-month follow-up study. Int Forum Allergy Rhinol. 2021;11(8):1249-52.

22. Nguyen NN, Hoang VT, Lagier JC, Raoult D, Gautret P. Long-term persistence of olfactory and gustatory disorders in COVID-19 patients. Clin Microbiol Infect. 2021;27(6):931-2.

23. Paderno A, Mattavelli D, Rampinelli V, Grammatica A, Raffetti E, Tomasoni M, et al. Olfactory and Gustatory Outcomes in COVID-19: A Prospective Evaluation in Nonhospitalized Subjects. Otolaryngol Head Neck Surg. 2020;163(6):1144-9.

24. Panda S, Mohamed A, Sikka K, Kanodia A, Sakthivel P, Thakar A, et al. Otolaryngologic Manifestation and Long-Term Outcome in Mild COVID-19: Experience from a Tertiary Care Centre in India. Indian J Otolaryngol Head Neck Surg. 2020;73(1):1-6.

25. Printza A, Katotomichelakis M, Metallidis S, Panagopoulos P, Sarafidou A, Petrakis V, et al. The clinical course of smell and taste loss in COVID-19 hospitalized patients. Hippokratia. 2020;24(2):66-71.

26. Printza A, Katotomichelakis M, Valsamidis K, Metallidis S, Panagopoulos P, Panopoulou M, et al. Smell and Taste Loss Recovery Time in COVID-19 Patients and Disease Severity. J Clin Med. 2021;10(5).

27. Rashid RA, Zgair A, Al-Ani RM. Effect of nasal corticosteroid in the treatment of anosmia due to COVID-19: A randomised double-blind placebo-controlled study. Am J Otolaryngol. 2021;42(5):103033.

28. Salmon Ceron D, Bartier S, Hautefort C, Nguyen Y, Nevoux J, Hamel AL, et al. Self-reported loss of smell without nasal obstruction to identify COVID-19. The multicenter Coranosmia cohort study. J Infect. 2020;81(4):614-20.

29. Samimi Ardestani SH, Mohammadi Ardehali M, Rabbani Anari M, Rahmaty B, Erfanian R, Akbari M, et al. The coronavirus disease 2019: the prevalence, prognosis, and recovery from olfactory dysfunction (OD). Acta Otolaryngol. 2021;141(2):171-80.

30. Sheng WH, Liu WD, Wang JT, Chang SY, Chang SC. Dysosmia and dysgeusia in patients with COVID-19 in northern Taiwan. J Formos Med Assoc. 2021;120(1 Pt 2):311-7.

31. Singh CV, Jain S, Parveen S, Deshmukh P. The outcome of fluticasone nasal spray on anosmia and triamcinolone oral paste in taste dysgeusia in COVID-19 patients. Am J Otolaryngol. 2021;42(4):103009.

32. Stavem K, Ghanima W, Olsen MK, Gilboe HM, Einvik G. Persistent symptoms 1.5-6 months after COVID-19 in non-hospitalised subjects: a population-based cohort study. Thorax. 2021;76(4):405-7.

33. Vacchiano V, Riguzzi P, Volpi L, Tappatà M, Avoni P, Rizzo G, et al. Early neurological manifestations of hospitalized COVID-19 patients. Neurol Sci. 2020;41(8):2029-31.

34. Vargas-Gandica J, Winter D, Schnippe R, Rodriguez-Morales AG, Mondragon J, Escalera-Antezana JP, et al. Ageusia and anosmia, a common sign of COVID-19? A case series from four countries. J Neurovirol. 2020;26(5):785-9.

35. Yen YF, Lai HH, Chan SY, Su VY, Chiu TF, Huang CY, et al. Olfactory disorder in patients infected with SARS-CoV-2. J Microbiol Immunol Infect. 2020.

**Letter**

1. Bénézit F, Le Turnier P, Declerck C, Paillé C, Revest M, Dubée V, et al. Utility of hyposmia and hypogeusia for the diagnosis of COVID-19. Lancet Infect Dis. 2020;20(9):1014-5.

2. Besharat S, Amlashi FI, Jahanshahi M, Besharat M. Anosmia in the era of COVID-19. Jundishapur Journal of Microbiology [Internet]. 2020; 13:[e103998 p.].

3. Chiesa-Estomba CM, Lechien JR, Saussez S. [The alteration of smell and taste in COVID-19 patients. A diagnostic resource in primary care]. Aten Primaria. 2020;52(8):592-3.

4. Costa K, Carnaúba ATL. Smell and taste disorders: warning signs for SARS-CoV-2 infection. Braz J Otorhinolaryngol. 2020;86(4):393-4.

5. Daval M, Corré A, Palpacuer C, Housset J, Poillon G, Eliezer M, et al. Efficacy of local budesonide therapy in the management of persistent hyposmia in COVID-19 patients without signs of severity: A structured summary of a study protocol for a randomised controlled trial. Trials. 212020. p. 666.

6. Fasunla AJ, Ibekwe TS. Sudden olfactory and gustatory dysfunctions: Important red flags in COVID-19. Niger J Clin Pract. 23. India2020. p. 1030-2.

7. Galougahi MK, Ghorbani J, Bakhshayeshkaram M, Naeini AS, Haseli S. Olfactory Bulb Magnetic Resonance Imaging in SARS-CoV-2-Induced Anosmia: The First Report. Acad Radiol. 2020;27(6):892-3.

8. Garavello W, Galluzzi F. Olfactory Dysfunction in COVID-19. Otolaryngol Head Neck Surg. 2020;163(1):182.

9. Gautier JF, Ravussin Y. A New Symptom of COVID-19: Loss of Taste and Smell. Obesity (Silver Spring). 282020. p. 848.

10. Gelardi M, Trecca E, Cassano M, Ciprandi G. Smell and taste dysfunction during the COVID-19 outbreak: a preliminary report. Acta Biomed. 2020;91(2):230-1.

11. Ghiasvand F, SeyedAlinaghi S. Isolated Anosmia as a Presentation of COVID-19: An Experience in a Referral Hospital. Infect Disord Drug Targets. 20. United Arab Emirates2020. p. 350.

12. Hall A, Frauenfelder C, Butler C, Coyle P, Hopkins C. Paediatric olfactory dysfunction: a chance to detect COVID-19? Arch Dis Child. 106. England2021. p. e17.

13. Hopkins C, Vaira LA, De Riu G. Self-reported olfactory loss in COVID-19: is it really a favorable prognostic factor? Int Forum Allergy Rhinol. 102020. p. 926.

14. Keyhan SO, Fallahi HR, Cheshmi B. Dysosmia and dysgeusia due to the 2019 Novel Coronavirus; a hypothesis that needs further investigation. Maxillofac Plast Reconstr Surg. 422020. p. 9.

15. Kheok SW, Teo NWY. Olfactory Loss of Function as a Possible Symptom of COVID-19. JAMA Otolaryngol Head Neck Surg. 146. United States2020. p. 873-4.

16. Lechien JR, Hoch CC, Vaira LA, Saussez S. The interest of fluticasone nasal spray in COVID-19 related anosmia is still not demonstrated. Am J Otolaryngol. 2021;42(4):103008.

17. Lechien JR, Hsieh J, Barillari MR, Cammaroto G, Hans S, Chiesa-Estomba CM, et al. Patient-Reported Outcome Questionnaires for the evaluation of olfactory and gustatory dysfunctions in COVID-19. Eur Arch Otorhinolaryngol. 2772020. p. 2393-4.

18. Lechner M, Patel ZM, Philpott C, Lund VJ. Olfactory Loss of Function as a Possible Symptom of COVID-19. JAMA Otolaryngol Head Neck Surg. 146. United States2020. p. 872-3.

19. Liou JM, Chen MJ, Hong TC, Wu MS. Alteration of taste or smell as a predictor of COVID-19. Gut. 702021. p. 806-7.

20. Locatello LG, Gallo O. Long-term recovery from COVID-19 anosmia: Promising findings and unanswered questions. J Intern Med. 2902021. p. 462-3.

21. Malnic B, Glezer I. Olfactory Loss of Function as a Possible Symptom of COVID-19. JAMA Otolaryngol Head Neck Surg. 146. United States2020. p. 872.

22. Mariño-Sánchez F, Santamaría-Gadea A, de Los Santos G, Alobid I, Mullol J. Psychophysical olfactory testing in COVID-19: is smell function really impaired in nearly all patients? Int Forum Allergy Rhinol. 102020. p. 951-2.

23. Nakashima T, Suzuki H, Teranishi M. Olfactory and gustatory dysfunction caused by SARS-CoV-2: Comparison with cases of infection with influenza and other viruses. Infect Control Hosp Epidemiol. 422021. p. 113-4.

24. Nasir S, Iftikhar PM. Association of COVID-19 with Anosmia and Hypogeusia. Am J Med Sci. 2020;360(4):414.

25. Otte MS, Klussmann JP, Luers JC. Persisting olfactory dysfunction in patients after recovering from COVID-19. J Infect. 2020;81(3):e58.

26. Ralli M, Di Stadio A, Greco A, de Vincentiis M, Polimeni A. Defining the burden of olfactory dysfunction in COVID-19 patients. Eur Rev Med Pharmacol Sci. 2020;24(7):3440-1.

27. Singh CV, Jain S, Parveen S. The outcome of fluticasone nasal spray on anosmia and triamcinolone oral paste in dysgeusia in COVID-19 patients. Am J Otolaryngol. 2021;42(3):102892.

28. Vaira LA, Salzano G, De Riu G. The importance of olfactory and gustatory disorders as early symptoms of coronavirus disease (COVID-19). Br J Oral Maxillofac Surg. 2020;58(5):615-6.

29. Vaira LA, Salzano G, Deiana G, De Riu G. Anosmia and Ageusia: Common Findings in COVID-19 Patients. Laryngoscope. 2020;130(7):1787.

30. Varricchio A, La Mantia I, Brunese FP, Ciprandi G. Smell recovery in patients with COVID-19: an experience with nebulized nasal treatment. J Biol Regul Homeost Agents. 35. Italy2021. p. 683-6.

31. Wan YM, Deng X, Tan EK. Olfactory dysfunction and COVID-19. Lancet Psychiatry. 2020;7(8):663.

32. Xydakis MS, Dehgani-Mobaraki P, Holbrook EH, Geisthoff UW, Bauer C, Hautefort C, et al. Smell and taste dysfunction in patients with COVID-19. Lancet Infect Dis. 2020;20(9):1015-6.

**Protocol**

1. Anosmia Rehabilitation in Patients Post Coronavirus Disease (COVID 19) [Internet]. Available from: https://ClinicalTrials.gov/show/NCT04374474.

2. Convalescent Plasma to Limit SARS-CoV-2 Associated Complications [Internet]. Available from: https://ClinicalTrials.gov/show/NCT04373460.

3. Coronavirus Smell Therapy for Anosmia Recovery [Internet]. Available from: https://ClinicalTrials.gov/show/NCT04422275.

4. Olfactory Training for Olfactory Dysfunction After Coronavirus Disease - 19 (COVID-19) [Internet]. Available from: https://ClinicalTrials.gov/show/NCT04764981.

5. Smell in Covid-19 and Efficacy of Nasal Theophylline [Internet]. Available from: https://ClinicalTrials.gov/show/NCT04789499.

6. Trial Evaluating the Efficacy of Local Budesonide Therapy in the Management of Hyposmia in COVID-19 Patients Without Signs of Severity [Internet]. Available from: https://ClinicalTrials.gov/show/NCT04361474.

7. Visual-OLfactory Training in Participants With COVID-19 Resultant Loss of Smell [Internet]. Available from: https://ClinicalTrials.gov/show/NCT04710394.

8. Effectiveness of Hypertonic saline nasal irrigation and gargling in COVID-19 [Internet].

9. Effect of Corton on olfactory dysfunction in COVID-19 patients [Internet]. Available from: https://en.irct.ir/trial/48379.

10. Effect of Lavender syrup on COVID-19 [Internet]. Available from: https://en.irct.ir/trial/48013.

11. The effect of olfactory training and vitamin A in the olfactory loss of patients with covid-19 [Internet]. Available from: https://en.irct.ir/trial/54178.

**Non laboratory confirmed COVID patients included**

1. Coelho DH, Kons ZA, Costanzo RM, Reiter ER. Subjective Changes in Smell and Taste During the COVID-19 Pandemic: A National Survey-Preliminary Results. Otolaryngol Head Neck Surg. 2020;163(2):302-6.

2. Hopkins C, Surda P, Kumar N. Presentation of new onset anosmia during the COVID-19 pandemic. Rhinology. 2020;58(3):295-8.

3. Hopkins C, Surda P, Vaira LA, Lechien JR, Safarian M, Saussez S, et al. Six month follow-up of self-reported loss of smell during the COVID-19 pandemic. Rhinology. 2021;59(1):26-31.

4. Hopkins C, Surda P, Whitehead E, Kumar BN. Early recovery following new onset anosmia during the COVID-19 pandemic - an observational cohort study. J Otolaryngol Head Neck Surg. 2020;49(1):26.

5. Reiter ER, Coelho DH, Kons ZA, Costanzo RM. Subjective smell and taste changes during the COVID-19 pandemic: Short term recovery. Am J Otolaryngol. 2020;41(6):102639.

6. Schwab J, Jensen CD, Fjaeldstad AW. Sustained Chemosensory Dysfunction during the COVID-19 Pandemic. ORL J Otorhinolaryngol Relat Spec. 2021;83(4):209-18.

7. Walsh-Messinger J, Kaouk S, Manis H, Kaye R, Cecchi G, Meyer P, et al. Standardized Testing Demonstrates Altered Odor Detection Sensitivity and Hedonics in Asymptomatic College Students as SARS-CoV-2 Emerged Locally. medRxiv. 2020.

**Language other than English, French, Spanish**

1. Bocksberger S, Wagner W, Hummel T, Guggemos W, Seilmaier M, Hoelscher M, et al. [Temporary hyposmia in COVID-19 patients]. Hno. 2020;68(6):440-3.

2. Haldrup M, Johansen MI, Fjaeldstad AW. [Anosmia and ageusia as primary symptoms of COVID-19]. Ugeskr Laeger. 2020;182(18).

3. Namazova-Baranova LS, Karkashadze GA, Zelenkova IV, Baranov AA, Vishneva EA, Kaytukova EV, et al. Non-randomized comparative study of olfaction in post-COVID-19 children. Intermediary results2020; 17(6):[502-7 pp.].

**Duplicate**

1. Iannuzzi L, Salzo AE, Angarano G, Palmieri VO, Portincasa P, Saracino A, et al. Gaining Back What Is Lost: Recovering the Sense of Smell in Mild to Moderate Patients After COVID-19. Chem Senses. 2020;45(9):875-81.

2. Lechner M, Liu J, Counsell N, Ta NH, Rocke J, Anmolsingh R, et al. Course of symptoms for loss of sense of smell and taste over time in one thousand forty-one healthcare workers during the Covid-19 pandemic: Our experience. Clin Otolaryngol. 462021. p. 451-7.

**Diagnosis method for COVID-19 not specified**

1. Mohamad SA, Badawi AM, Mansour HF. Insulin fast-dissolving film for intranasal delivery via olfactory region, a promising approach for the treatment of anosmia in COVID-19 patients: Design, in-vitro characterization and clinical evaluation. Int J Pharm. 2021;601:120600.
