# Supplementary material for: Olfactory recovery following infection with COVID-19: A systematic review
Source: PLoS One. 2021 Nov 9;16(11):e0259321. doi: 10.1371/journal.pone.0259321 (PMC8577770; doi:10.1371/journal.pone.0259321)
Supplement: S3 Text — (DOCX) [file pone.0259321.s008.docx]

**OLFACTORY RECOVERY FOLLOWING INFECTION WITH COVID – 19:**

**A SYSTEMATIC REVIEW PROTOCOL FOR PROSPERO**

**Review Questions**

**Primary:**

To what extent does olfactory recovery occur in patients reporting olfactory dysfunction related to infection with COVID-19?

**Secondary:**

What is the time duration for recovery from COVID-19 related olfactory dysfunction?

What patient characteristics increase the risk of olfactory dysfunction related to COVID-19 infection? What patient characteristic increase the likelihood of recovery of olfactory dysfunction related to COVID-19 infection?

What therapies have been tried to manage olfactory dysfunction associated with COVID-19 infection, and which have been shown to improve olfactory recovery?

**Literature Searches**

Ovid Medline, Embase, Cochrane Central and LitCovid. Systematic Reviews on this topic will be retrieved from Medline and their references will be scanned for additional eligible studies.

Search strategy:

1 (exp coronavirus/ or coronavirus*.mp. or corona virus*.mp.) and (wuhan or beijing or shanghai or hubei).mp. (4965)

2 ((coronavirus* or corona virus* or coronavirus* or coronaviridae or coronaviridae or betacoronavirus*) adj3 ("19" or "2019")).tw. (18765)

3 covid.tw,kw. (62718)

4 covid19.tw,kw. or covid 19.kw. (24060)

5 sars cov 2.tw,kw. (20494)

6 (ncov or n cov).tw,kw. (1852)

7 (novel coronavirus* or novel corona virus*).tw,kw. (7621)

8 (CoV 2 or CoV2 or sarscov2 or 2019nCoV or novel CoV or wuhan virus*).tw,kw. (19725)

9 (Coronavirus Infections/ or Severe Acute Respiratory Syndrome/) and (Pandemics/ or pandemic*.tw,kf.) (14884)

10 or/1-9 (73344)

11 Olfaction Disorders/ or smell/ or Sensation Disorders/ (67731)

12 smell*.tw,kw. (27282)

13 olfact*.tw,kw. (115398)

14 anosmi*.tw,kw. (7161)

15 (dysosmi* or Hyposmi*).tw,kw. (4409)

16 or/11-15 (172675)

17 10 and 16 (833)

18 exp animals/ not humans/ (17565360)

19 17 not 18 (444)

20 19 use medall (422) Medline

21 (Coronavirinae/ or coronavirus*.mp. or corona virus*.mp.) and (wuhan or beijing or shanghai or hubei).mp. (4937)

22 ((coronavirus* or corona virus* or coronavirus* or coronaviridae or coronaviridae or betacoronavirus*) adj3 ("19" or "2019")).tw. (18765)

23 (covid or covid19).tw. (62032)

24 sars cov 2.tw. (17232)

25 (ncov or n cov).tw. (1625)

26 (novel coronavirus* or novel corona virus*).tw. (7140)

27 (CoV 2 or CoV2 or sarscov2 or 2019nCoV or novel CoV or wuhan virus).tw. (18023)

28 (coronavirus infection/ or severe acute respiratory syndrome/) and (pandemic/ or pandemic*.tw.) (19218)

29 limit 28 to yr="2019 -Current" (18119)

30 21 or 22 or 23 or 24 or 25 or 26 or 27 or 29 (71933)

31 exp smelling disorder/ (11207)

32 (smell* or olfact*).tw. (130124)

33 (anosmi* or dysosmi* or Hyposmi*).tw. (9554)

34 or/31-33 (137354)

35 30 and 34 (865)

36 (exp animal/ or nonhuman/) not exp human/ (11917689)

37 35 not 36 (862)

38 37 use emczd (463)

39 (exp coronavirus/ or coronavirus*.mp. or corona virus*.mp.) and (wuhan or beijing or shanghai or hubei).mp. (4965)

40 ((coronavirus* or corona virus* or coronavirus* or coronaviridae or coronaviridae or betacoronavirus*) adj3 ("19" or "2019")).tw. (18765)

41 covid.tw,kw. (62718)

42 covid19.tw,kw. or covid 19.kw. (24060)

43 sars cov 2.tw,kw. (20494)

44 (ncov or n cov).tw,kw. (1852)

45 (novel coronavirus* or novel corona virus*).tw,kw. (7621)

46 (CoV 2 or CoV2 or sarscov2 or 2019nCoV or novel CoV or wuhan virus*).tw,kw. (19725)

47 (Coronavirus Infections/ or Severe Acute Respiratory Syndrome/) and (Pandemics/ or pandemic*.tw.) (14879)

48 or/39-47 (73343)

49 Olfaction Disorders/ or smell/ or Sensation Disorders/ (67731)

50 smell*.tw,kw. (27282)

51 olfact*.tw,kw. (115398)

52 anosmi*.tw,kw. (7161)

53 (dysosmi* or Hyposmi*).tw,kw. (4409)

54 or/49-53 (172675)

55 48 and 54 (833)

56 55 use cctr (5)

57 20 or 38 or 56 (890)

58 remove duplicates from 57 (525)

59 58 use medall (412)

60 58 use emczd (108)

61 58 use cctr (5)

**Types of study to be included (Eligibility criteria)**

We will include interventional studies (i.e., RCT, non-RCT, controlled before/after, interrupted time series) and observational studies (i.e., prospective or retrospective cohort, case-control, case series, cross-sectional) reporting olfactory dysfunction in patients infected by COVID-19 with data on treatment or recovery of olfaction.

Our eligibility criteria for inclusion and exclusion criteria are defined as following:

**Inclusion criteria**

1. Studies with an interventional or observational design, that report data on patients with olfactory dysfunction due to COVID-19 infection and report data on olfaction measured by any objective olfactory test (see list below), Likert scale and/or visual analog scale (VAS).

| **TEST** |
| --- |
| 12-item Odour Memory Test |
| Biolfa olfactory test |
| Connecticut Chemosensory Clinical Research Center Test (CCCRC) |
| Cross-Cultural Smell Identification Test (CC-SIT) |
| Odorant Confusion Matrix |
| Olfactory Perception Threshold Test |
| Pocket Smell Test (PST) |
| San Diego Odour Identification Test |
| Scandinavian Odour Identification Test |
| Smell Diskettes Test |
| Smell Threshold Test |
| Sniff Magnitude Test |
| Sniffin’Sticks |
| T&T Olfactometer |
| University of Pennsylvania Smell Identification Test (UPSIT) |

1. Observational or experimental design studies that report data on length of time to recovery for olfaction dysfunction.
2. Observational or experimental design studies that report data on the extent of recovery of olfactory dysfunction measured by any objective olfactory test (see list) or by VAS.

**Exclusion criteria**

1. Review articles, case reports, letter to the editor, editorials, commentaries, abstracts
2. Studies not published in English, Spanish, or French
3. Non-human studies

**Condition or domain being studied**

Current or recovery of olfactory dysfunction (the partial or complete loss of olfaction) related to COVID-19 infection.

**Participants/population**

Patients diagnosed with olfactory dysfunction related to positive COVID-19 infection.

**Intervention(s), exposure(s)**

Treatment of COVID-19 positive patients with olfactory dysfunction and the therapies used. Due to the nature of the research question, studies involving or not involving treatments of olfactory dysfunction will be of interest, so long as data related to olfactory outcomes are reported.

**Comparator(s)/control**

No control group will be required. For studies having a control group (such as RCTs), stratifications of data for reporting and quantitative syntheses may be considered to represent changes in olfactory dysfunction.

**Context**

Hospitals, primary care (e.g., general practitioner, walk-in clinics), pharmacists in any country.

**Main outcome(s)**

Olfactory change as a result of therapy for olfactory dysfunction due to COVID-19 infection as documented by objective measures of olfaction (eg. e.g. UPSIT, Sniffin’ Sticks).

**Additional outcome(s)**

- Interventions or therapies used for management of olfactory dysfunction in patients with COVID-19 infection.
- Risk/protective factors for the development and/or resolution of olfactory dysfunction associated with COVID-19 infection.

**Process of Study Selection**

Based on the inclusion criteria previously specified, initial screening of title and/or abstract (if available) will be performed by one evaluator. Any exclusions will be confirmed by a second person. The full texts of the selected studies identified as potentially relevant based upon review of titles/abstracts will be obtained, and final ascertainment of eligibility will be performed independently by two evaluators. Disagreements will be resolved by consensus discussion and consultation of a third party (if needed). A PRISMA flow diagram will be presented to summarize the numbers of studies identified, included and excluded (and the reasons for exclusion). Study selection will be managed using Covidence systematic review software.

**Data Collection**

One reviewer will independently extract and record the data using a standardized data extraction form implemented in Covidence. A second reviewer will independently verify 20% of studies for general characteristics and 100% for all other information. Discrepancies will be settled by consensus and discussion amongst the reviewers. The following items will be extracted from each included study:

1. Identification: Study ID (if available), citation, contact author details, country of conduct of research origin
2. Methods: study design, total study duration, study location(s), sample size, stated study objective, approach to data analysis
3. Participants: mean (SD) patient age (or median/range if mean unavailable), race/ethnicity, diagnostic method for COVID-19. Patient setting (non-hospitalized, hospitalized, medical care requiring intubation), severity clinical manifestation of COVID-19 infection (as reported in the study).
4. Outcomes: objective measures of olfactory dysfunction and recovery. Timing for evaluation of olfactory dysfunction; scoring and interpretation of the instrument. Therapies used for treatment of COVID-19 or the olfactory loss specifically. Risk/protective factors associated with development of olfactory dysfunction associated with COVID-19, and/or its resolution.
5. Results: Measures of olfaction dysfunction according to each instrument at each time point. Length of time to recovery. Listings of treatments administered to treat olfactory dysfunction. Association measures for risk/protective factors for the development and/or resolution of olfactory dysfunction.
6. Key conclusions of the study authors
7. Funding

**Risk of Bias (quality assessment)**

The Cochrane Risk of Bias tool will be used for RCTs and the Newcastle Ottawa Scale will be used for cohort and case-control studies to assess risk of bias as appropriate. Case series, cross-sectional studies, controlled before/after studies and interrupted time series will be evaluated using appropriate tools from the Joanna Briggs Institute. Two independent reviewers will independently evaluate the risk of bias or methodological quality of the eligible studies. Disagreements will be resolved by consensus.

**Strategy for data synthesis**

We will present study characteristics in tables and/or text in the final report. A descriptive summary of the results for each of the included studies will be provided. We will address the primary and secondary questions of interest as follows:

- Duration of olfactory dysfunction: Meta-analysis of outcomes may be performed subject to quantity, quality and clinical homogeneity of studies using random effects meta-analysis. Should meta-analysis not be feasible (due to heterogeneity between studies or a lack of data), we will conduct a narrative analysis of studies. If data from studies are reported incompletely, we will contact authors twice over 3 weeks by email to seek additional information. Several instruments are specific to olfaction dysfunction (e.g. UPSIT, Sniffin’ Sticks). It is expected that included studies studying comparisons of different interventions will report treatment effect measures based upon different instruments. Therefore, the summary statistic used to combine treatment effects will be the standardized mean difference (SMD); if treatment effects are reported using the same scale, the summary statistic will be the weighted mean difference (WMD). In cases of non-comparative analyses (e.g. for time duration of dysfunction), pooled averages will be calculated. For all measures of effect, the 95% Confidence Interval (CI) will be calculated. Review Manager Software and Comprehensive Meta-Analyst will be used for all quantitative analyses. Statistical heterogeneity will be evaluated, stratified by study design, using the I^2^ test. An I^2^ higher than 50% will be considered substantial. Reasons for statistical heterogeneity will be explored using subgroup and sensitivity analysis. If appropriate, data will be pooled and random effects model will be used.
- Treatments used for management of olfactory dysfunction: A descriptive summary of the types of interventions used (including schedule, dose and other relevant information) will be provided, along with a summary of the range of reported benefits.
- Risk/protective factors for olfactory dysfunction: a descriptive summary of risk/protective factors assessed for impact on the risk and resolution of COVID-19 associated olfactory dysfunction will be presented. This will include methods of measurement and analysis used within the included studies.

**Analysis of subgroups or subsets**

Subgroups of interest for analysis will include geographic region, age less than 60 years of age, sex, specific treatment measures, severity of olfactory loss, duration of olfactory loss prior to onset of treatment, patient comorbidities, severity of COVID-19 infection (non-hospitalised, hospitalised, medical treatment requiring intubation). Other criteria may be considered depending on the data available. Sensitivity analysis may be undertaken, where needed, regarding the risk of bias (restricting to studies at low risk of bias), data issues and measurement of outcomes, or to explore other unanticipated sources of heterogeneity. All subgroup analyses that are feasible for conduct will be reported in the completed review, and those conducted post-hoc will be clearly identified as such.

We will evaluate analysis for funnel plot asymmetry, where needed and feasible (at least 10 studies).^1,2^

The quality of evidence for all primary outcomes and adverse events will be assessed using the GRADE framework.

**Contact details for further information**

Dr. Shaun Kilty

[skilty@toh.ca](mailto:skilty@toh.ca)

**Organisational affiliation of the review**

The Department of Otolaryngology – Head and Neck Surgery

University of Ottawa

Ottawa, Canada

**Review team members and their organisational affiliations**

**Ali Jafar, MD**. Department of Otolaryngology - Head and Neck surgery. University of Ottawa

**Andrea Lasso, MSc.** Ottawa Hospital Research Institute (OHRI), Ottawa, Canada

**Brian Hutton, MSc, PhD.** Director, Knowledge Synthesis Unit, Ottawa Hospital Research Institute (OHRI), School of Epidemiology, Public Health and Preventive Medicine, University of Ottawa.

**Candyce Hamel, MSc, PhD (candidate).** Senior Clinical Research Associate, OHRI Knowledge Synthesis Group.

**Shaun Kilty, MD.** Department of Otolaryngology - Head and Neck Surgery. University of Ottawa. Ottawa Hospital Research Institute (OHRI).

**Type and method of review**

Systematic review, Meta-analysis.

**Anticipated or actual start date**

August 15, 2020

**Anticipated completion date**

September 30, 2020

**Funding sources/sponsors**

None

**Conflicts of interest**

None

**Language**

English

**Country**

Canada

**References**

(1) Sterne JAC, Egger M. Funnel plots for detecting bias in meta-analysis: Guidelines on choice of axis. Journal of Clinical Epidemiology 2001; 54: 1046-1055.

(2) Sterne JAC, Egger M, Moher D, Boutron I (editors). Chapter 10: Addressing reporting biases. In: Higgins JPT, Churchill R, Chandler J, Cumpston MS (editors), Cochrane Handbook for Systematic Reviews of Interventions version 5.2.0 (updated June 2017), Cochrane, 2017. Available from www.training.cochrane.org/handbook].
