# Supplementary material for: Olfactory recovery following infection with COVID-19: A systematic review
Source: PLoS One. 2021 Nov 9;16(11):e0259321. doi: 10.1371/journal.pone.0259321 (PMC8577770; doi:10.1371/journal.pone.0259321)
Supplement: Attachment — Submitted filename: Response to Reviewers.docx [file pone.0259321.s009.docx]

Sep 26, 2021

Re: PONE-D-21-25736

Olfactory recovery following infection with COVID-19: A systematic review

Eng Ooi
Academic Editor
PLOS ONE

Thank you for your email from Sep 14, 2021 regarding the submission of our manuscript “Olfactory recovery following infection with COVID-19: A systematic review”. We appreciate the time taken to review our work.

Please see below our answer to the comments and questions raised during the review.

1. **Please ensure that your manuscript meets PLOS ONE's style requirements, including those for file naming. The PLOS ONE style templates can be found at
   https://journals.plos.org/plosone/s/file?id=wjVg/PLOSOne_formatting_sample_main_body.pdf and** 
   [**https://journals.plos.org/plosone/s/file?id=ba62/PLOSOne_formatting_sample_title_authors_affiliations.pdf**](https://journals.plos.org/plosone/s/file?id=ba62/PLOSOne_formatting_sample_title_authors_affiliations.pdf)

**Response:** Thank you for the comment. We have formatted the manuscript document to follow PLOS ONE’s style requirements.

2. **Please review your reference list to ensure that it is complete and correct. If you have cited papers that have been retracted, please include the rationale for doing so in the manuscript text, or remove these references and replace them with relevant current references. Any changes to the reference list should be mentioned in the rebuttal letter that accompanies your revised manuscript. If you need to cite a retracted article, indicate the article’s retracted status in the References list and also include a citation and full reference for the retraction notice.**

**Response:** Thank you for the comment. We have checked the references included in this paper and to the best of our knowledge none have been retracted.

3. **We note that you have indicated that data from this study are available upon request. PLOS only allows data to be available upon request if there are legal or ethical restrictions on sharing data publicly. For more information on unacceptable data access restrictions, please see http://journals.plos.org/plosone/s/data-availability#loc-unacceptable-data-access-restrictions.**

**In your revised cover letter, please address the following prompts:**

a) **If there are ethical or legal restrictions on sharing a de-identified data set, please explain them in detail (e.g., data contain potentially sensitive information, data are owned by a third-party organization, etc.) and who has imposed them (e.g., an ethics committee). Please also provide contact information for a data access committee, ethics committee, or other institutional body to which data requests may be sent.**
b) **If there are no restrictions, please upload the minimal anonymized data set necessary to replicate your study findings as either Supporting Information files or to a stable, public repository and provide us with the relevant URLs, DOIs, or accession numbers. For a list of acceptable repositories, please see http://journals.plos.org/plosone/s/data-availability#loc-recommended-repositories.**

**We will update your Data Availability statement on your behalf to reflect the information you provide.**

**Response:** Thank you for the comment. All the data required to replicate the results of this study is available as part of the supporting information.

**REVIEWERS COMMENTS:**

**Reviewer #1:** This is an in-depth systematic review looking at the recovery of olfactory dysfunction in patients with COVID – 19. I only have a few comments:

**1. The number of records excluded/irrelevant, don’t match the text (page 8, line 194) and figure 1. In the text it is 2533, in figure 1 it is 2546. Is this an error?**

**Response:** Thank you for your comment. The correct number is 2546, we have corrected this error.

**2. Page 18, line 255 – what is the range of sample size? There is only 1 number: 1363, please correct**

**Response:** Thank you for your comment. This error has been corrected. The correct range is 7-1363 patients

**3. The authors mentioned the rates of full recovery of olfaction. Was there any data that could be extracted about the rates of partial recovery or some degree of recovery?**

**Response:** Thank you for your comment. Tables 4 and 5 show the proportion of patients who reported anosmia or hyposmia at the end of the follow up period. If studies reported data on partial improvement of no improvement/recovery this is specified for each study on these columns.

**4. Were papers including children specifically excluded from the review?**

**Response:** Thank you for your question. The inclusion criteria for this review did not specifically exclude studies that included children. However, none of the included studies was conducted in pediatric population.

**Again a great paper and I commend the authors on the very thorough review.**

**Reviewer #2:** Thank you for the opportunity to review this systematic review on an important topic.
Overall, this is a well conducted systematic review with a priori methods published.
You have clearly reported the search and study selection with independent reviewers to reduce bias.
Good use of JBI tools for study quality analysis. Descriptive analysis is appropriate as significant heterogeneity in study design and results.

**1. There is a lot of good data presented in all the tables, however, they are all very busy. To allow easier perusal for reader, please reduce unnecessary words and use acronyms where appropriate.**

**Response:** Thank you for your comments. We have edited the tables for better visualization and ensured that acronyms were used consistently.

**2. Table 1 and Table 2 – “Not reported” in table 1, “NR” in table 2. Maintain consistent acronyms.
Please use one or the other**

**Response:** Thank you for your comment. We have updated to tables and “NR” has been used consistently.

**3. Table 3. Lechien 2021 (58) – Sample size. Reduce wording. Ie. 233 (Objective) 1362 (Subjective)**

**Response:** Thank you for your comment. We have updated Table 3 to include your suggestion.

**4. Study population
• Can you clarify the definition of mild vs mild-moderate symptom severity?**

**Response:** Thank you for your comment. The study population section has been updated to clarify the definition of mild and moderate disease severity.

**5. Line 298. The studies identified to have one test evaluating OD at a single point in time were deemed to have high level of bias. Please clarify in this section if these studies were excluded from analysis. As it would not be possible obtain follow up data with only one data point.**

**Response:** Thank you for your comment. These studies were not included. The section Extent and time to recovery has been updated to clarify this point.

**6. Table 4. The “Time of evaluation” column is very busy. Can this be simplified? For example.
• Test 1: Day 1.
• Test 2: Day 10.
• Test 3: Day 31**

**Response:** Thank you for your comment. We have updated Table 4 and 5. We have also eliminated the Timing of evaluation in Tables 1-3 as this was the same information contained in Tables 4-5.

**7. Table 5. If you could label “Time of evaluation” column the same as Table 4. Similar to table 4, simplify and reduce the wording in this column. This will make the follow up times easier to follow for the reader.**

**Response:** Thank you for your comment. We have made the suggested change.

**Reviewer #3:** This paper adequately summarises the growing number of investigations into COVID-19 related olfactory dysfunction, and provides succinct evidence to counsel patients on the expected recovery of olfactory dysfunction. The article is technically sound, and the data supports the conclusions made.
The methods were appropriate, and the PRISMA reporting guideline for systematic reviews was followed.
All supplementary data was made available for review
There are no concerns regarding the methods, reporting of results, or discussion.

**Response:** Thank you for your comments.

Please do not hesitate to contact me with any questions.

Sincerely,

Shaun Kilty MD, FRCSC

Otolaryngology- Head & Neck Surgery / Otorhinolaryngologie et Chirurgie Cervico-faciale

Full Professor

Department of Otolaryngology-Head and Neck Surgery

The University of Ottawa and The Ottawa Hospital

Senior Clinician Investigator

Ottawa Hospital Research Institute (OHRI)
